# Income‐Related Inequalities in Future Health Prospects

**DOI:** 10.1002/hec.4965

**Published:** 2025-04-18

**Authors:** Gustav Kjellsson, Dennis Petrie, Tom Van Ourti

**Affiliations:** ^1^ Department of Economics School of Public Health & Communicy Medicine University of Gothenburg Gothenburg Sweden; ^2^ Centre for Health Governance, Department of Economics University of Gothenburg Gothenburg Sweden; ^3^ Centre for Health Economics Monash University Melbourne Australia; ^4^ Erasmus Centre for Health Economics Rotterdam Erasmus University Rotterdam Rotterdam the Netherlands; ^5^ Erasmus School of Health Policy and Management Erasmus University Rotterdam Rotterdam the Netherlands; ^6^ Erasmus School of Economics Erasmus University Rotterdam Rotterdam the Netherlands; ^7^ Tinbergen Institute Erasmus University Rotterdam Rotterdam the Netherlands

**Keywords:** concentration index, health inequality, risk

## Abstract

Measuring health disparities is key to monitoring health systems, but hitherto disparities in the individual risk people face about their future health has been neglected. This paper integrates individual health risk into income‐related health inequality measurement. We develop a rank dependent health inequality index that considers inequalities in each individual's expected future health and the dispersion of their future health prospects. It is useful when a social planner wants to account for risk averse preferences in the assessment of income‐related inequalities of future health prospects. The empirical application using Australian longitudinal data highlights that neglecting individual risk underestimates income‐related inequalities in future health prospects since the poor not only face worse expected future health, but also faced greater dispersion in their future health prospects compared to the rich.

## 1 Introduction

Health disparities are systematically related to income, although to varying degrees across space and time (Wagstaff and van Doorslaer 2000; Cutler et al. 2012; Deaton 2013; Chetty et al. 2016). The concentration index is the main health economics workhorse for measuring these disparities. It captures the extent of health differences across individuals ranked by an indicator of socioeconomic status such as income (Wagstaff et al. 1991; O’Donnell et al. 2008, 2016). The concentration index has been used to report cross‐sectional estimates of health inequalities across countries (van Doorslaer et al. 1997), over time (Gravelle and Sutton 2003; Wagstaff et al. 2003), or how health dynamics are related to the income position of individuals (Jones and Nicolas 2004; Van Ourti et al. 2009; Allanson et al. 2010; Coveney et al. 2020; Allanson and Petrie 2021). However, inequality in the individual risk people face about their future health has received no attention in the literature on measurement of health inequality.^1^ In this paper we address this topic.

The literature on decisions under risk stresses that an individuals' valuation (or anticipated utility) of particular lotteries often depends on the risk involved (Quiggin 1982; Starmer 2000). The riskiness of an individual's future health prospects might thus impact on the value the individual places on it, and it may therefore be relevant to analyze how individual health risk varies across the population. When individual health risk is unequally distributed across income, a social planner—who acknowledges that individuals are on average risk‐averse—may recognize the dispersion of future health prospects as an additional source of income‐related health inequalities on top of disparities in the expected level of future health.

In this paper, we develop an index of income‐related inequalities in risky health prospects. The index can be calculated in two steps. First, the social planner values each individual's distribution of risky future health prospects. These valuations are increasing in expected future health and decreasing in the dispersion of future health prospects faced by that individual (Shalit and Yitzhaki 1984); and thus are sensitive to the trade‐off between improvements in expected future health and reductions in the dispersion of future health. In the second step, the social planner summarizes the co‐variation of the individual‐specific valuations across current income using the generalized concentration index (Wagstaff et al. 1991; Erreygers 2009). It will indicate pro‐rich inequalities when the rich face higher expected future health compared to the poor, and become more pro‐rich if the rich additionally face lower dispersion in their future health prospects. An alternative interpretation of the index considers the income‐related inequality in each quantile of each individual's health distribution, from everybody's worst possible future health outcome (the lowest health quantile) all the way up to the income‐related inequality in their best possible health outcome (the highest health quantile). If the inequality in each health quantile is given equal weight then the index collapses to the generalized concentration index which ignores individual risk, while giving more weight to inequalities at lower compared to higher health quantiles is compatible with the trade‐off that emerges under risk‐aversion. The aggregation from the lowest up to the highest health quantile further spells out that the index incorporates individual health risk (and not societal risk), that is focus is on how distributions of risky future health prospects, faced by each individual, vary by current income.

We derive the risk‐sensitive inequality index under the assumption that the social planner is able to predict individual‐specific distributions of future potential health outcomes. This includes the different potential health levels that might emerge, as well as the probabilities of each future health level faced by every individual. Our social planner takes an ex‐ante viewpoint and does not care about the health level that the individual will eventually experience in the future once individual risk is resolved. This assumption also exemplifies our explicit focus on the impact of *individual* risk for health inequality ex‐ante (today), rather than the impact of *societal* risk for the extent of future health inequality ex‐post.^2^ The social planner accounts for risk aversion by imposing the same valuation function onto the distribution of risky health prospects of each individual. Finally, we assume throughout the paper that the social planner considers inequality to be unchanged for absolute changes in each health prospect across all individuals, but the framework can be extended to other inequality invariance stances (Kolm 1976; Erreygers and Van Ourti 2011; Allanson and Petrie 2013; Kjellsson et al. 2015).

This paper makes two contributions to the literature on health inequality measurement. First, we extend the standard inequality measurement apparatus used by health economists such that it can account for risky health prospects. The approach is intuitive, has two equivalent interpretations and allows policy makers to monitor how individual health risk varies with an indicator of socioeconomic status such as current income. The second contribution lies in combining the newly developed measurement apparatus with data from the Australian Household, Income and Labor Dynamics in Australia (HILDA) Survey. We compare inequalities in future health prospects across three sub‐populations based on country of birth—born in Australia, born overseas in another English‐speaking country, and born overseas in a non‐English speaking country. This is of public policy relevance since Australia has a particularly large migrant population and differences in language, culture and limited knowledge of the Australian healthcare system may put them at higher risk of poorer future health prospects (Jatrana et al. 2014). We find that income‐related inequalities in the dispersion of future health prospects contribute substantially to the overall index in all three sub‐populations, even after standardizing for age which is the most important predictor of mortality and future health. This finding confirms the importance of considering the additional inequalities that arise from dispersion, on top of those arising from expected future health. Our empirical results additionally illustrate that inequalities in the dispersion of future health were most concentrated among those born overseas, while inequality in expected future health was largest among those born overseas in a non‐English speaking country.

The remainder of this paper is organized as follows. The methods of inequality measurement of risky future health prospects are laid out in Sections 2 and 3. The empirical illustration is discussed in Section 4 and Section 5 concludes.

## 2 Measuring Income‐Related Inequalities Without Risk

The standard approach to the measurement of income‐related health inequalities does not consider individual risk, and is based on the standard concentration index (Wagstaff et al. 1991) and its rank‐dependent generalizations (Wagstaff 2005; Erreygers 2009; Erreygers et al. 2012; Kjellsson et al. 2015; O’Donnell et al. 2016). In this paper, we use the generalized concentration index $I\left(H,Y\right)$ which measures absolute health inequalities:

(1) $$I\left(H,Y\right)=\frac{1}{n}\sum_{i=1}^{n}z_{i}h_{i}$$

where $H=\left[h_{1},h_{2},\text{…},h_{n}\right]$ and $Y=\left[y_{1},y_{2},\text{…},y_{n}\right]$ are vectors with deterministic health and income levels. Individuals are ranked by income $\left(y_{1}\leq y_{2}\leq \text{…}\leq y_{n}\right)$, and $z_{i}=\left(2i-1-n\right)/n$ is a social weight that linearly increases with income rank from $\left(1-n\right)/n$ for the poorest individual to $\left(n-1\right)/n$ for the richest individual, and equals zero for the individual with median income.

The welfare properties of the generalized concentration index are well known (Mehran 1976; Wagstaff et al. 1991; Bleichrodt and van Doorslaer 2006; Erreygers and Van Ourti 2011). The index records no health inequality when everyone experiences the same level of health. Concentration of health among the poor leads to negative values of the index (pro‐poor) and positive values indicate pro‐rich inequalities. The index satisfies the principle of income‐related health transfers which imposes that health inequality becomes more pro‐poor after a health‐transfer from a richer to a poorer person (Bleichrodt and van Doorslaer 2006); and $I\left(H,Y\right)$ measures absolute inequalities and thus imposes that inequality remains unchanged when everyone's health changes by the same absolute amount.

**FIGURE 1:**
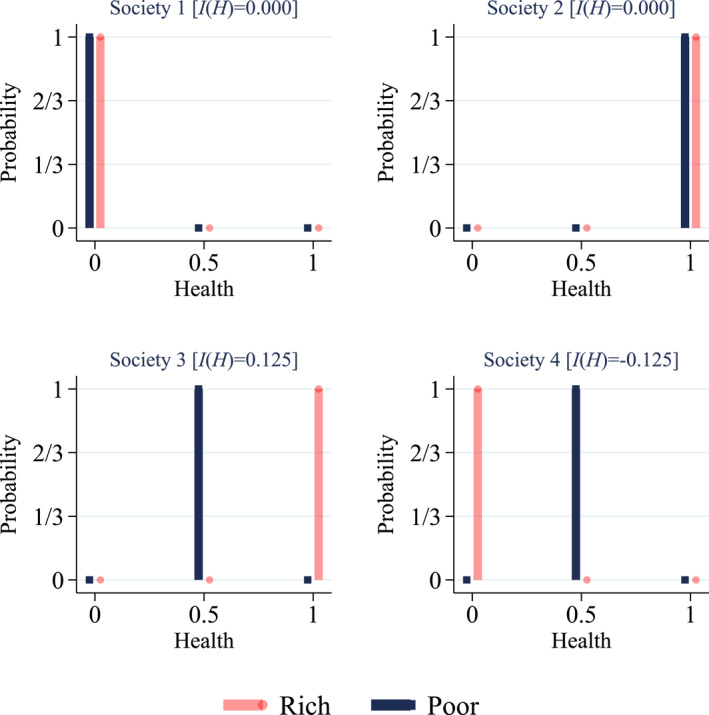
Hypothetical societies without health risk: Probability density functions.

In a scenario without individual risk, the generalized concentration index can be used to evaluate income‐related inequalities in health. Consider Figure 1 which shows four examples of hypothetical two‐person societies where individuals face no individual risk, that is with a probability of 100% they are either dead (0), in fair (0.5) or perfect health (1). In society 3, the rich individual is in perfect health and the poor individual in fair health resulting in pro‐rich inequalities as health differences favor the rich. Society 4 is an example of inequalities in favor of the poor (pro‐poor), while there are no income‐related health inequalities in a society where everyone is dead (society 1) or in perfect health (society 2).

## 3 Introducing Individual Health Risk Into the Measurement Apparatus

When individuals face risk, one has to consider the full distribution of each individual's future health prospects (from their worst future health quantile to their best future health quantile) and how these individual‐specific distributions vary by income. This section first discusses the social planner's valuation of the distribution of future potential health outcomes for each individual in isolation. Next, it presents the between‐individual inequality in these valuations by income and examines the implicit value judgments of the resulting index. We end the section with an alternative, but equivalent, derivation of the index that first calculates income‐related inequalities across individuals for each future health quantile, and next aggregates these inequalities across quantiles, thereby further clarifying that the index incorporates *individual*, and not *societal* health risk.

### 3.1 Valuing Risky Future Health Prospects

For a given future period, each individual faces a distribution of potential future health outcomes. We assume that the social planner is able to predict Q quantiles of this distribution (based on individual characteristics). Denote the qth future health quantile of individual i as $h_{i}^{q}$, so that $h_{i}^{1}\leq h_{i}^{2}\leq \text{…}\leq h_{i}^{Q}$ for q=1,2,…,Q. The social planner values (in the current period) individual i’s distribution of potential future health outcomes using a rank‐dependent utility function (Quiggin 1982):

(2) $$V_{i}=\frac{1}{Q}\sum_{q=1}^{Q}w_{q}h_{i}^{q}$$

and uses the same quantile weights $w_{q}$ for every individual. The quantile weights are positive, decrease from the worst health quantile to the best health quantile, and sum to Q, that is $\left(1/Q\right)\sum_{q=1}^{Q}w_{q}=1$. $V_{i}$ thus increases more when a future health quantile in the lower part of individual i’s health distribution (a worse health prospect) improves compared to the same improvement in the upper part of the distribution (a better health prospect).

The extent to which the social planner incorporates that individuals appreciate health, and are risk‐averse is determined by the functional form of the quantile weights.^3^ Assuming the weights to be $w_{q}=\left(2Q-2q+1\right)/Q$, that is strictly positive (satisfying Pareto) and linearly declining with the health quantile Q (reflecting risk‐aversion), implies that $V_{i}$ corresponds to the certainty equivalent level of future health and penalizes expected health—which does not depend on the quantile weights—by the generalized Gini index of the health quantiles $D_{i}$—which does depend on the quantile weights. The generalized Gini index is a standard measure of dispersion: for the same expected level of health, more dispersed future health quantiles lead to a larger penalty (Shalit and Yitzhaki 1984). This can also be seen from adding and subtracting individual i’s expected health, $\bar{h_{i}}$, to Equation (2):

(3) $$V_{i}=\frac{1}{Q}\sum_{q=1}^{Q}w_{q}\left(h_{i}^{q}+\bar{h_{i}}-\bar{h_{i}}\right)=\bar{h_{i}}-\frac{1}{Q}\sum_{q=1}^{Q}h_{i}^{q}\left(1-w_{q}\right)=\bar{h_{i}}-D_{i}$$

### 3.2 Income‐Related Inequality in the Valuation of Risky Future Health Prospects

The social planner worries that the poor face less favorable distributions of future health prospects compared to richer individuals, and therefore replaces the deterministic health realizations $h_{i}$ in Equation (1) by the valuation function $V_{i}$. The resulting index equals the weighted sum of all future health quantiles across all individuals with the weights equaling the product of the income and quantile weights^4^ ^:^

(4) $$I\left(V\right)=\frac{1}{n}\sum_{i=1}^{n}z_{i}V_{i}=\frac{1}{n}\frac{1}{Q}\sum_{i=1}^{n}\sum_{q=1}^{Q}z_{i}w_{q}h_{i}^{q}$$

and where dependence of $I\left(V,Y\right)$ on Y is suppressed for notational simplicity.

Combining Equations (3) and (4) shows that income‐related inequalities in future health prospects can be decomposed into income‐related inequality in expected future health $I\left(\bar{H}\right)$ and income‐related inequality in the dispersion of future health prospects $I\left(D\right)$:

(5) $$I\left(V\right)=\frac{1}{n}\sum_{i=1}^{n}z_{i}\bar{h_{i}}-\frac{1}{n}\sum_{i=1}^{n}z_{i}\left[\frac{1}{Q}\sum_{q=1}^{Q}h_{i}^{q}\left(1-w_{q}\right)\right]=I\left(\bar{H}\right)-I\left(D\right)$$

Since $I\left(\bar{H}\right)$ and $I\left(D\right)$ uncover separate but complementary features of income‐related inequality in future health prospects, both indices might indicate opposite patterns with respect to current income (pro‐poor vs. pro‐rich inequalities). In such instances, the decomposition of the overall index I(V) in Equation (5) reveals whether inequality in expected future health outweighs that in the dispersion of future health prospects, or vice versa.^5^

Equation (5) further illustrates the respective roles and normative meaning of the income weights $z_{i}$ and quantile weights $w_{q}$. As the income weights appear in $I\left(\bar{H}\right)$ and $I\left(D\right)$, the implicit normative concern about how future health prospects vary with current income is similar for both sub‐components of the overall index. The quantile weights, instead, only appear in $I\left(D\right)$ and do not matter for inequalities in expected future health; and thus determine the trade‐off between the two sub‐components of the overall index.

Both weights also determine the bounds of the overall index in Equation (4), as well as the bounds of the two components in Equation (5). For a health variable bounded between 0 and 1, such as in our empirical application, the overall index attains its maximum (minimum) value of 0.25 (− 0.25) when expected future health of all individuals above (below) the median income rank equals full (minimum) health. The bounds of the inequality index of expected health are the same as the overall index, whereas the inequality index for the dispersion of future health prospects ranges from − 0.0625 to 0.0625; the latter maximum is reached when the richest 50% of the population have certain future health and the poorest 50% of the population have a 50% chance of full health and 50% chance of zero health.^6^ These narrower bounds restrict the possible fraction of total inequalities that may be due to inequalities in the dispersion of future health prospects, and reflect the normative implications of linear weights for the trade‐off between inequalities in expected future health prospects and their dispersion.

**FIGURE 2:**
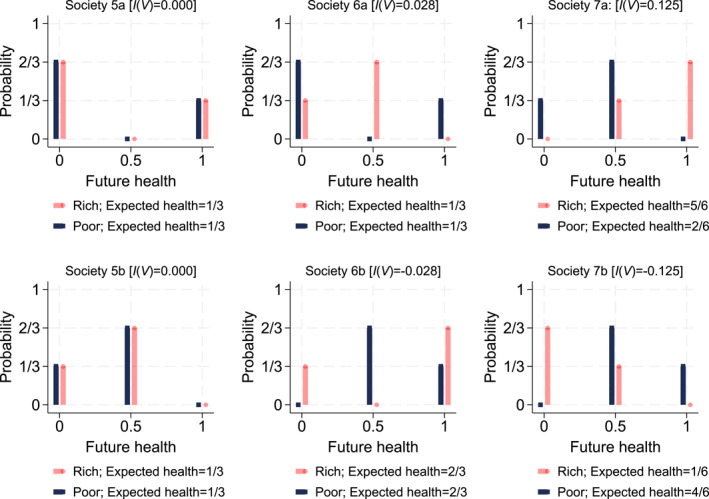
Hypothetical societies with individual health risk: Probability density functions.

Figure 2 provides a graphical illustration of our approach by adding individual risk to the hypothetical societies in Figure 1 such that individuals face two (out of three) possible future health levels with a non‐zero probability. There is no income‐related inequality in society 5a: even though the poor and rich individual face risky future health prospects, they face the same individual risk and hence there are no disparities across income. Society 5b depicts a similar situation but both individuals face lower dispersion in future health prospects. In societies 6, the poor and rich experience the same expected future health level, but face different dispersion in future health. In 6a, disparities are pro‐rich because the richer individual faces less dispersion, while the opposite pattern occurs in society 6b. In societies 7, expected health differs for the poor and rich individual. Therefore—based on expected future health—disparities are in favor of the rich in society 7a and in favor of the poor in society 7b. In both societies, rich and poor face the same dispersion of future health since we use the generalized Gini index which is a measure of absolute inequality: the health prospects of the rich individual can be obtained by adding (7a) or subtracting (7b) 0.5 units of health from the prospects of the poor individual. Thus, dispersion does not add to income‐related inequality in societies 7.

While the linear income and quantile weights have been chosen to make the connection with well‐known indices in the literature—the generalized concentration index (linear income weights, Equation 1) and the generalized Gini index (linear quantile weights, Equation 3)—the framework is compatible with non‐linear weights (see Section 3.3.2 and appendix Appendix A). Non‐linear weights allow putting more or less weight on particular parts of the income distribution (Wagstaff 2002; Erreygers et al. 2012) and/or the distribution of future health prospects (Bleichrodt and van Doorslaer 2006). This has no implications for Equations (3), (4), (5), except for the third equality sign in Equation (3) and the second equality sign in Equation (5) which will no longer hold as the trade‐off between inequalities in expected future health and the dispersion of future health prospects depends on the functional form chosen for the quantile weights. Similarly, applying other functional forms for the weights will also lead to different ranges of possible fractions that the dispersion sub‐component can take as a part of the overall index.

### 3.3 Inequality Impact of Health Quantile Changes

This section discusses the index's response to (a) one individual experiencing a change in one particular health quantile; and (b) mean preserving transfers between different health quantiles of different individuals.

#### 3.3.1 Changing One Health Quantile of One Individual

In case one individual experiences an increase in one quantile of her future health distribution, the sign of the income weight $z_{i}$—that is whether the individual is relatively poor or rich—determines whether the inequality change is pro‐poor or pro‐rich, with more positive (negative) weights leading to larger inequality changes in favor of the rich (poor). The quantile weights do not affect the sign of the inequality change and only matter for its magnitude (as does the size of the health transfer). As quantile weights are positive and linearly declining with q, improvements in the worst possible future health outcomes will have a larger impact compared to improvements in already good possible future health outcomes of the same individual.

#### 3.3.2 Mean‐Preserving Transfers

The impact of a mean‐preserving health transfer from a health quantile of one individual to a health quantile of another individual, or between health quantiles of the same individual, depends on the income and quantile weights of the involved individual(s) (and the size of the transfer). Transfers between health quantiles of the same individual (or between individuals with the same income) lead to more pro‐poor (pro‐rich) inequalities when the individual is poor (rich) and the transfer reduces the dispersion of the future health outcomes (i.e. $D_{i}$ is reduced). The opposite pattern occurs when the dispersion is increased. Health transfers between health quantiles of individuals with different incomes will generally be pro‐poor when health is donated from a health quantile of a richer individual to a health quantile of a poorer individual; while donations from a poorer to richer individual will generally be pro‐rich. However, while a health transfer from rich to poor always induces a pro‐poor change in a setting without risk,^7^ this does not necessarily translate to a situation with individual risk because the value placed on the health transfer may differ based on the quantiles involved. We discuss these exceptions, including potentially non‐linear income and quantile weights in appendix Appendix A.

### 3.4 Alternative Interpretation of the Index

The index in Equation (4) can also be obtained by first estimating income‐related inequalities across individuals for each health quantile $I\left(H^{q}\right)$, and next obtaining the final index $I\left(V\right)$ as a weighted sum of these quantile‐specific income‐related inequalities using the quantile weights:

(6) $$I\left(V\right)=\frac{1}{nQ}\sum_{i=1}^{n}\sum_{q=1}^{Q}z_{i}w_{q}h_{i}^{q}=\frac{1}{Q}\sum_{q=1}^{Q}w_{q}\left(\frac{1}{n}\sum_{i=1}^{n}z_{i}h_{i}^{q}\right)=\frac{1}{Q}\sum_{q=1}^{Q}w_{q}I\left(H^{q}\right)$$

Expressing the index in this format explicitly illustrates how inequalities in each quantile are valued differently depending on its location in the distribution. Given the restrictions imposed on $w_{q}$, more weight is given to income‐related inequalities at lower health quantiles and lesser weight to inequalities in quantiles in the upper part of the health distributions. Equation (6) also reveals that the index incorporates individual, but not societal, risk as the index aggregates across quantiles and not “states of the world.” The index indeed considers how the covariance between the q‐th future health quantile and current income varies across quantiles; not on how the covariance between realized future health and current income varies across “states of the world.”

**FIGURE 3:**
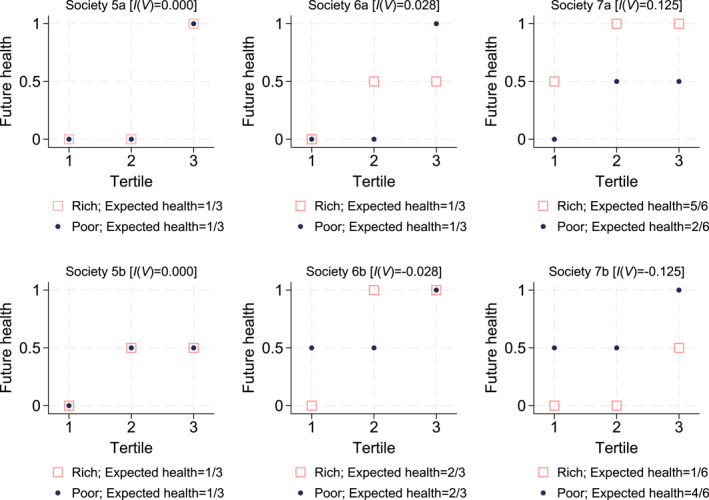
Hypothetical societies with individual health risk: Quantile functions.

This alternative derivation can be graphically illustrated using the quantile functions (inverse distribution functions) of the risky health prospects faced by the currently rich and poor. Figure 3 repeats the hypothetical societies in Figure 2 using quantile functions rather than PDF's. For example, the second health tertile of the currently poor in society 6a equals 0, while that of the currently rich individual equals 0.5; hence inequalities in the second health tertile favor the rich. At tertile 3, inequalities favor the poor, while there are no income‐related disparities in the first tertile. The overall conclusion about the extent of disparities in risky future health prospects then depends on the importance given to the separate tertile‐specific disparities. In the context of risk aversion, more importance is given to the lower tertiles; and hence society 6a has pro‐rich disparities in risk. A similar reasoning can be applied to the other societies in Figure 3, and the resulting estimates of income‐related disparities will be identical to those obtained from the first calculation approach in Figure 2.

## 4 Empirical Illustration

The overall inequality measure in Equation (4) assumes that the social planner is able to observe each individual's distribution of health prospects for some future period. In reality, however, the social planner will only be able to observe the realization of future health once individual risk is resolved. Thus, the empirical version of our ex‐ante approach needs to be predicted. To do this we can use the distribution of observed realizations for similar individuals, as defined by their observable characteristics, as an approximation of the health distribution individuals would have faced. We could also extrapolate into the future for individuals alive today based on the individual risk estimated for similar individuals in the past. This approach aligns with other papers that also estimate risk, such as Flores and O'Donnell (2016) who estimate exposure to the risk of catastrophic medical payment. Like Flores and O'Donnell (2016) we use quantile regressions conditioned on a set of covariates to predict individuals' future health distribution (e.g., Koenker and Bassett 1978). Since our objective is to predict each individual's health distribution based on their observable covariate set, it is the conditional and not the unconditional quantiles that are of interest here. These can be predicted using quantile regressions. Note that the covariates should be interpreted as predictors of an individual's future health distribution, and including potentially endogenous predictors still provides meaningful predictions for the social planner of the future health quantiles for each individual. If the social planner could observe additional important predictors of an individuals future health quantiles then the social planner would face less uncertainty and be better informed about the risk individuals face. While the data we use in our empirical example includes a rich set of predictors of future health prospects, one can not rule out that our estimates deviate from those that would be obtained under full information.

### 4.1 Data

We use the Household, Income and Labor Dynamics in Australia (HILDA) annual survey. HILDA is a household‐based panel study in Australia, which covers a large national representative sample of Australian households occupying private dwellings and includes a wide range of topics (Summerfield et al. 2014). Wave 1 (2001) consisted of 7682 households and 19,914 individuals and this was boosted in 2011 with a top up sample. Our illustration uses the sample who answered the full questionnaire in 2011 and considers their health prospects in 2019 including whether they were reported to have died before then.^8^ The income variable used in this study is the equivalized household income in 2011 which accounts for the number of adults and children in the household using the OECD equivalence scale.^9^ Our health measure is derived in terms of Quality Adjusted Life Year (QALY) weights from the SF6D and ranges from zero to one, where dead individuals are assigned a value of zero.^10^

The covariate set used in the predictive model includes individual characteristics in 2011: health (QALY weight), equivalized household income (expressed as the natural logarithm of 2019 Australian dollars in thousands), age (and age squared), sex, highest education qualification, unemployment status, marital status, smoking status, dummy for being a risky drinker, and immigrant status. Table 1 shows the descriptive statistics of these K covariates.

**TABLE 1:** Descriptives.

Variables (n = 12,833)	Mean	Std. Dev.
Health 2011	0.75	0.12
Health 2019*	0.70	0.22
Equivalized household income ($'000s per annum)**	54.98	30.49
Age (years old)	43.03	17.28
Male	47%	
Education		
Less than secondary school completion (reference)	28%	
Secondary school completion	13%	
Post school certificate or diploma	32%	
University degree or higher	26%	
Unemployed	3%	
Marital status		
Single (reference)	16%	
Married or living with partner	70%	
Divorced/Separated/Widowed	14%	
Smoking status		
Never (reference)	51%	
Current	19%	
Former	30%	
Risky drinker***	6%	
Country of birth		
Australia [Aus born] (reference)	76%	
Other English speaking country [OS Eng]	11%	
Non‐English speaking country [OS non‐Eng]	13%	

*Note:* Our sample excludes 16.4% (2525 out of 15,358) of people who are dropped due to missing health or other explanatory factors in 2011: mostly due to the SF36 not being fully completed (2444 did not complete the SF36 as this appears in the self completion part of the survey rather than the telephone component). * The health average for 2019 is based on 9690 observations since, in 2019, only 9052 (74%), of the 12,195 alive report their health and zero health is assigned to the 638 deceased (5% of the 12,833). ** The 12,833 observations also excludes the 0.5% poorest of the population in 2011 to remove negative incomes for which the natural logarithm of income is not defined and symmetrically we also removed the 0.5% richest. *** Risky drinker is defined as drinking at least on average once per week and having more than 6 standard drinks on a usual occasion.

### 4.2 Predicting Future Health Prospects

The nature of the health variable has implications on how to predict future health prospects. In our application, we need to consider that health ranges from 0 to 1 where the minimum value 0 indicates death, but the minimum value observed conditional on survival is approximately 0.3. To account for the extreme outcome, death, and restrict the possible predicted health quantiles to fall between the bounds of 0.3 and 1 when they are predicted to be alive, we estimate a two‐part model (Duan et al. 1983) for health prospects in 2019 conditional on a set of initial (2011) characteristics $x_{ik}$.

The first part predicts the probability of being alive in 2019, (i.e., $h_{i}>0$) using a standard logit:

(7) $$P\left(h_{i}>0\left|x_{i1},\text{…},x_{iK}\right.\right)=\frac{\exp\left(\alpha_{0}+\sum_{k=1}^{K}\alpha_{k}x_{ik}\right)}{1+\exp\left(\alpha_{0}+\sum_{k=1}^{K}\alpha_{k}x_{ik}\right)}$$

To restrict the predicted health quantiles to lie within the bounds of 0.3 and 1 we use a logistic quantile regression conditional on survival (Bottai et al. 2010; Orsini and Bottai 2011). In practice, we transform the health outcome onto a continuous scale between positive and negative infinity^11^ using a logistic transformation $g\left(h_{i}\right)=\ln\left(\frac{h_{i}-0.3}{1.001-h_{i}}\right)$, and then use this transformed variable as the dependent variable in a linear quantile regressions (conditional on survival):

(8) $$Q_{g\left(h_{i}\right)}\left(q\left|h_{i}>0,\;x_{i1},\text{…},x_{iK}\right.\right)=\psi_{0}^{q}+\sum_{k=1}^{K}\psi_{k}^{q}x_{ik}$$

We estimate Equation (8) for q=0.01,0.02,…0.99,^12^ and re‐transform the predicted quantiles (on the continuous scale between negative and positive infinity) back onto the bounded scale as^13^ ^:^

(9) $${\hat{h}}_{i}^{q}={\hat{Q}}_{h}\left(q\left|h_{i}>0,\;x_{i1},\text{…},x_{iK}\right.\right)=\frac{1.001exp\left({\hat{\psi }}_{0}^{q}+\sum_{k=1}^{K}{\hat{\psi }}_{k}^{q}x_{ik}\right)+0.3}{1+\exp\left({\hat{\psi }}_{0}^{q}+\sum_{k=1}^{K}{\hat{\psi }}_{k}^{q}x_{ik}\right)}$$

These predictions are used jointly with the individuals' predicted mortality risk from the logit model, $\hat{P}\left(mortality\right)=1-{\hat{p}}_{i}=1/\left(1+\exp\left(\hat{\alpha }+\sum_{k=1}^{K}{\hat{\alpha }}_{k}x_{ik}\right)\right)$ to obtain the distribution of possible future health outcomes (including death). We generate an approximation of the predicted future health distribution by drawing 1000 observations for each individual from the predictions of the two parts of the model.^14^ By probability $\hat{P}\left(mortality\right)=1-{\hat{p}}_{i}$ these draws are assigned the value of 0, and by probability $\hat{P}\left(alive\right)={\hat{p}}_{i}$ these are drawn from the predictions of the 99 quantiles in Equation (9). These draws are then ordered such that ${\hat{h}}_{i}^{1}\leq {\hat{h}}_{i}^{2}\leq \text{⋯}\leq {\hat{h}}_{i}^{1000}$ provide the predicted 1000 quantiles for each individual's future health prospects.

### 4.3 Age‐Standardizing Inequality in Health Prospects

To interpret inequalities in future health prospects, it is useful to complement the index I(V) with an age‐standardized version of the index that accounts for differences in the age structure of the population. Because age is by far the most important predictor of mortality (see Section 4.4.1), a non‐standardized index will be dominated by the variation of age across income.^15^

Assuming the Q quantiles for each individual i can be expressed as a linear function of each individual's characteristics in the initial period, one can use Q OLS regressions (one for each quantile) to estimate (ˆ suppressed to simplify notation)^16^

(10) $$h_{i}^{q}=\beta_{0}^{q}+\beta_{\mathrm{age}}^{q}x_{i,age}+\beta_{age^{2}}^{q}x_{i,age^{2}}+\sum_{k=3}^{K}\beta_{k}^{q}x_{ik}.$$

Substituting Equation (10) into the overall inequality index in Equation (4) yields

(11) $$I\left(V\right)=I\left(age\right)\left(\frac{1}{Q}\sum_{q=1}^{Q}w_{q}\beta_{\mathrm{age}}^{q}\right)+I\left(age^{2}\right)\left(\frac{1}{Q}\sum_{q=1}^{Q}w_{q}\beta_{age^{2}}^{q}\right)+\sum_{k=3}^{K}I\left(X_{k}\right)\left(\frac{1}{Q}\sum_{q=1}^{Q}w_{q}\beta_{k}^{q}\right)$$

Following the approach in Wagstaff et al. (2003), age‐standardized inequality is then obtained as $I\left(V\right)$ minus the contribution of age—which equals the product of the generalized concentration index of age, $I\left(age\right)$, and a weighted sum of the age coefficients in each quantile $\left(1/Q\right)\sum_{q=1}^{Q}w_{q}\beta_{\mathrm{age}}^{q}$—and the contribution of ${\text{age}}^{2}$ which is similarly defined. In the same fashion, one can remove the contribution of age from income‐related inequality in expected health and health dispersion (Equation 5) and quantile‐specific income related inequalities (Equation 6).

Equations (10) and (11) also inform on the impact of unobserved predictors on the value of the index. Adding an error term to the right hand side of Equation (10), representing the true deviation from the unknown distribution due to omission of unobservable predictors,^17^ shows that the impact of unobservable predictors will depend on the covariance between current income and the dispersion of the error term (generalized Gini across quantiles). Since this expression depends on the underlying data and the included set of predictors, its magnitude and sign, which ultimately determines the impact on the inequality ranking, can not be known a priori.^18^

### 4.4 Application to Income‐Related Health Inequalities Among Migrant Groups in Australia

Australia has a large share of inhabitants that were born overseas: in 2011 slightly more than 75% of the population was born in Australia and less than half of those born overseas originate from other English speaking countries (Table 1). The health and health inequalities in migrant groups versus those born in Australia is of particular public policy interest because migrants may face worse future health prospects due to language and cultural barriers (Jatrana et al. 2014). This, in addition to their reduced knowledge of the Australian healthcare system, may not only reduce their use of preventive healthcare services (e.g. screening programs) (Yeasmeen et al. 2020) but may also delay their use of acute healthcare services (Bhaskar et al. 2019). This is likely to expose them to more (individual) risk in their future health prospects. The Australian healthcare system operates as a mixed public‐private model where those with private insurance or willing or able to pay higher out‐of‐pocket costs can access healthcare with shorter wait times (Cheng 2014). This means that for migrants with higher incomes, these resources may help moderate the individual risk they face. Thus, understanding the income‐related health inequalities in future health prospects of different migrant groups compared to those born in Australia is of particular interest.

We predict future health prospects for all 12,833 individuals available in the 2011 wave of HILDA using the approach of Section 4.2. We then use these predictions to compare differences in income‐related inequalities in future health prospects across three sub‐populations: those born in Australia, those born overseas in a non‐English speaking country, and those born overseas in an English speaking country. As our main aim is empirical illustration of the new overall inequality measure in Equations (4), (5), (6), we use a simple approach: the predicted future health prospects are derived from one encompassing model for all 12,833 individuals, while the inequality estimates are calculated separately for each sub‐population using the approach from the previous section.

The estimates obtained from the encompassing model and the age‐standardized inequality results are presented in subsequent sections. We also graphically represent inequality as (1) penalising income‐related inequality in expected future health with income‐related inequality in the dispersion of future health (Equation 5), and as (2) the weighted sum of quantile‐specific income‐related inequalities (Equation 6).

#### 4.4.1 Mortality & Quantile Regressions

A snapshot of the regression results underlying the inequality estimates is provided in Appendix Table A2. We show the logit regression results for mortality by 2019 and the estimated coefficients for a sample of the quantile regressions (for quantiles at 5%, 25%, 50%, 75%, 95%) for health conditional on survival in 2019.^19^ The results from these regressions are used to simulate the expected health quantiles for each individual (unconditional on survival) for 2019 considering both mortality and their health quantiles conditional on survival.

#### 4.4.2 Income‐Related Inequality in Expected Future Health and the Dispersion of Future Health

We first consider, without age‐standardisation, how income (of 2011)‐related inequality in future health prospects in 2019 varies across the three migrant groups. Summary statistics for these three groups are presented in Appendix Table A3 and Table 2 shows that future health prospects are concentrated among the rich in all three sub‐populations, but most so among those born overseas in a non‐English speaking country. The estimated inequality levels in future health prospects are substantial, compared to the maximum inequality level of 0.25 and compared to the level of cross‐sectional inequality in 2011 (observed income and health levels in 2011) which was 0.0306, 0.0259 and 0.0270 for respectively those born in Australia, overseas English, and overseas non‐English.

**TABLE 2:** Inequality in future health prospects $I\left(V\right)$, expected future health $I\left(\bar{H}\right)$ and dispersion in future health $-I\left(D\right)$ in 2019 by 2011 incomes for those born in Australia (Aus born), overseas in an English‐speaking country (OS Eng), and overseas in a non‐English speaking country (OS non‐Eng).

	$I\left(V\right)$			Age‐standardized $I\left(V\right)$		
	Aus born	OS Eng	OS non‐Eng	Aus born	OS Eng	OS non‐Eng
Future health prospects	0.0410	0.0447	0.0457	0.0227	0.0218	0.0270
Due to expected health	0.0332	0.0354	0.0373	0.0195	0.0181	0.0234
Due to health dispersion	0.0078	0.0093	0.0084	0.0032	0.0037	0.0036
Sample size (*N*)	9783	1442	1608	9783	1442	1608

*Note:* $I\left(V\right)=I\left(\bar{H}\right)-I\left(D\right)$. The (minimum) maximum values of $I\left(V\right)$, $I\left(\bar{H}\right)$ and $-I\left(D\right)$ are respectively (−)0.25, (−)0.25 and (−)0.0625.

Age‐standardized inequalities are also informative as age is the most important predictor of mortality. Once we standardize for age, the overall inequality in future health prospects almost halves as older individuals tend to be poorer and have both worse expected future health (inequality due to expected health in Table 2 reduces after age‐standardization) and higher dispersion in the distribution of future health prospects (age‐standardization reduces inequality due to health dispersion). Table 2 also depicts the decomposition into the inequality in expected future health and the inequality in health dispersion in accordance with Equation (5). Across all three sub‐populations and both before and after age‐standardization, inequality due to health dispersion contributes to overall inequality in future health prospects and thus exacerbates the level of overall inequality compared to if one only considered the inequality in expected future health. The exact magnitude of this contribution depends on the assumption of linear quantile (and income) weights (see Section 3.2), and would be different if non‐linear weights would be used. Nevertheless, the fact that health dispersion contributes to inequalities in total future health prospects illustrates the empirical relevance of our approach in the Australian migrant setting.

**FIGURE 4:**
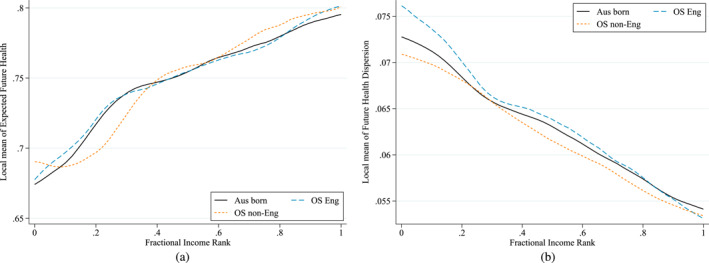
Age‐standardized expected future health and health dispersion by income rank (2019). Expected future health and health dispersion are calculated as in Equation (5) but evaluated at the average age of the entire Australian population in 2011. That is, substituting (10) into Equation (5) where age has been equalized to 43.03.

Figure 4a,b shed more light on how expected future health and health dispersion in 2019 vary by income rank in 2011. It shows expected future health and health dispersion predicted from Equation (10) after setting everyone's age equal to the average age of the entire Australian population (see Table 1). Since I(V), $I\left(\bar{H}\right)$ and I(D) measure absolute inequalities, one should compare the slope (and not the height) of expected future health and health dispersion with current income rank across the three sub‐populations. In line with Table 2, the slope of expected future health is most positive for those born overseas in a non‐English speaking country meaning that good expected future health is most concentrated among the rich in this sub‐population. While this does not hold among the poorest 20% (here the slope is flatter for the non‐English overseas), it is more than compensated by the more positive slope among the richest 80%. Figure 4b further confirms that future health dispersion is concentrated among the poor, but less so among those born in Australia as its slope is less negative.

**FIGURE 5:**
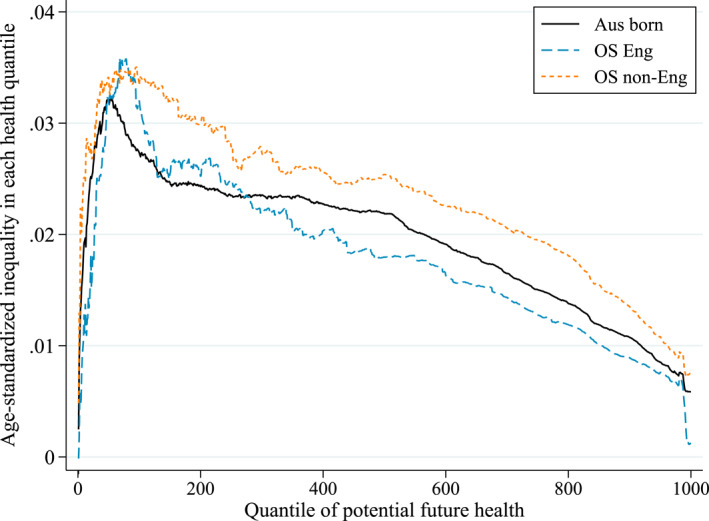
Age‐standardized inequality in prospective health outcomes per quantile. Quantile specific age‐standardized inequalities, measured as the generalized concentration index of each age‐standardized health quantile, is calculated following Equations (10) and (6), that is $\sum_{k=3}^{K}\beta_{k}^{q}I\left(X_{k}\right)$.

The overall index $I\left(V\right)$ can also be interpreted as the weighted sum of quantile‐specific income‐related inequalities. Its age‐standardized version is obtained after combining Equations (6) and (10) and removing the contribution of age, that is $\frac{1}{Q}\sum_{q=1}^{Q}w_{q}\left[\sum_{k=3}^{K}\beta_{k}^{q}I\left(X_{k}\right)\right]$. Figure 5 illustrates how the level of age‐standardized income‐related inequality in each of the 1000 predicted future quantiles $\sum_{k=3}^{K}\beta_{k}^{q}I\left(X_{k}\right)$ varies across the quantiles, where q=1 is the worst and q=1000 the best quantile for all individuals in 2019. Across all three sub‐populations, the age‐standardized quantile‐specific inequalities approach zero for the worst quantiles indicating a somewhat equal distribution of the worst health outcomes as all income groups face some future chance of dying in all sub‐populations. As we move up the quantiles the inequalities rapidly increase with the poor on average having increasingly worse potential future health outcomes in these lower quantiles compared to the rich. Inequalities are highest around percentiles 5 to 10, and then start decreasing down to a level of around 0.1 in the highest quantiles (as there is some chance of good future outcomes in all income groups across all sub‐populations). Compared to the two other sub‐populations, the overseas non‐English have higher age‐standardized income‐related inequalities at almost each quantile. Despite the Australian born facing lower age‐standardized inequalities between percentiles 5 to 25 than the overseas English, overall they face higher age‐standardized inequalities in future health prospects (see also Table 2).

We also explore the implications for income‐related inequality due to future health dispersion if the social planner could only observe a limited set of covariates to be used in the predictive model. We re‐estimated inequality due to future health dispersion along the lines of Equations (5), (6), (7), (8), (9), (10), (11) while excluding employment status, marital status, smoking status and alcohol status (and thus only including initial health, income, age, sex and education). We find only minor differences. For the age‐standardised results the income‐related inequality due to health dispersion is 0.0031 compared to 0.0032 for Australian born; 0.0046 versus 0.0037 for those born in an English speaking overseas country; and 0.0040 versus 0.0036 for those born in an non‐English speaking country. Importantly, the inequality ranking of the three sub‐populations is unaffected.

## 5 Discussion and Conclusion

This paper develops a framework to measure income‐related inequalities in risky future health prospects that considers not only inequalities in expected future health but also inequalities in the dispersion of individual's future health prospects. It complements existing approaches by integrating individual risk into the standard inequality measurement apparatus used by health economists.

We have then illustrated the usefulness of this framework by empirically considering differences in the inequality in future health prospects between three migrant sub‐populations in an Australian cohort. Our empirical study shows that poorer individuals are not only more likely to have worse health in the future, on average, but also more likely to face higher levels of dispersion in their future health prospects, thereby illustrating the importance of our approach. This result remains after standardizing for age. We also find that age‐standardized inequalities are highest among those born overseas in a non‐English speaking country and that the contribution of health dispersion is largest among those born overseas.

The overall inequality measure may be decomposed into inequalities in expected future health prospects and inequalities in the dispersion of the future health distribution. As the two components may evolve in opposite directions, we encourage empirical examination of both parts separately in addition to the overall index. It may, for example, be of importance for policy decisions whether the two parts of the index develop in the same or in opposite directions. The functional form of the quantile weights determines the implicit trade‐off between inequalities in expected future health and the dispersion of health. Future empirical applications may therefore also experiment with more flexible (non‐linear) weights.

This paper is, as far as we know, the first attempt to integrate individual risk into health inequality measurement. Our empirical approach is therefore primarily an illustration, and as such there is room for improvements. We used predictions (and not causal estimates) to estimate the conditional distribution of future health prospects and while this improves the accuracy of predicted future health prospects, it also means that age‐standardizing should not be given a causal interpretation; and rather be seen as a conditional association. A related point concerns omitted predictors in the quantile regressions. While our data has a rich set of covariates to predict future health prospects, clearly we do not observe all possible predictors. If the social planner had more observable characteristics with which to predict future health quantiles then the social planner would face less uncertainty and be better informed about how the individual risk individuals face varies by current income. The extent to which the additional information would impact direction and magnitude, and importantly, the ranking of the association between risk and current income can however not be known a priori. We therefore recommend checking the robustness of the inequality ranking across populations or time by re‐estimating the index with a different covariate set. We also applied the same overlapping model to predict future health prospects for all individuals in the three migrant groups; thereby imposing that the conditional associations between the predictors and respectively mortality and future health is the same in all three groups.

We have also imposed several assumptions which require scrutiny in future research. First, our approach focuses on inequalities in risky health prospects for one future point in time, and thereby neglects health dynamics and the risk in one's future stream of health. A natural future development would be to consider the dynamics of health inequalities in a longitudinal framework (Van Ourti et al. 2009; Allanson et al. 2010), and incorporate the expected stream of future health and the health risk associated with this stream of health (e.g. lifetime QALYs; duration analyses). Modeling these dynamics and assessing the related inequalities is more complex and also requires a long panel of rich individual data, and although we have such data at hand, these are in general rare. The fact that our measurement apparatus requires data at only two points in time therefore increases its usefulness considerably. However, estimating individual risk in future health prospects using observed differences in health across individuals does ignore the possible apriori risks associated with those states of the world that did not end up eventuating. Second, our framework considers how individual risk in future health is related to current income. A potential expansion of the framework would be to also acknowledge that also future income, and not only health, are uncertain. Future work accounting for uncertainty in income would likely have to leave the rank‐dependent framework and move into a framework allowing for flexible trade‐offs between uncertainty in income and health. Finally, our approach introduces individual risk into the measurement of income‐related health inequalities, capturing how distributions of risky future health prospects, faced by each individual, vary by current income. This ex‐ante approach could be replaced by ex‐post evaluation provided the empirical method to predict future health prospects captures aggregate, or societal, risk—the “states of the world”—such that the covariance between future realized health and current income would be allowed to differ between “states of the world.” We leave such an endeavor for future research.

## Conflicts of Interest

The authors declare no conflicts of interest.

## Figures and Tables

**FIGURE A1:**
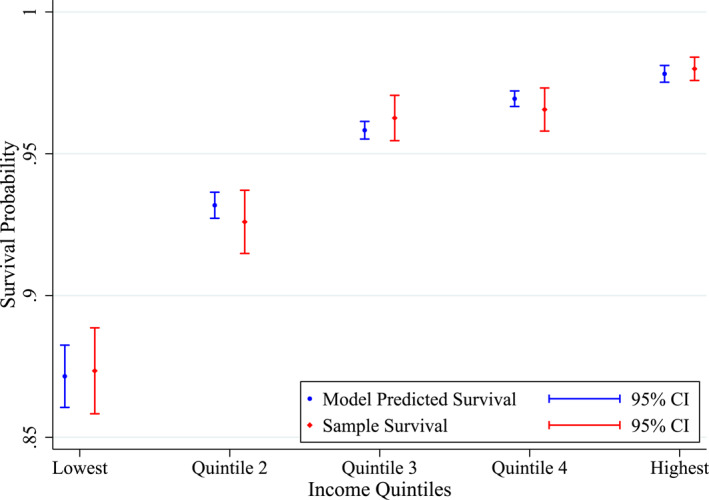
Model predictions and sample survival estimates by income quintile.

## Data Availability

This paper uses unit record data from the Household, Income and Labor Dynamics in Australia (HILDA) Survey. The HILDA Project was initiated and is funded by the Australian Government Department of Social Services (DSS) and is managed by the Melbourne Institute of Applied Economic and Social Research Melbourne Institute. The findings and views reported in this paper, however, are those of the authors and should not be attributed to either DSS or the Melbourne Institute.

## Endnotes

^1^
The role of risk (or insecurity) has been studied in the income inequality literature (e.g., Makdissi and Wodon 2003; Cruces et al. 2004; Bossert and D'ambrosio 2013; N. Rohde et al. 2014; Makdissi and Wodon 2018), but is intrinsically different from the case in this paper which focuses on the bivariate setting of risky future health prospects across income.

^2^
Diamond (1967), Broome (1982), Fleurbaey (2018) and Abásolo and Tsuchiya (2020), among others, discuss ex‐post versus ex‐ante social evaluation of risky states.

^3^
We have a broad concept of risk aversion in mind which reflects both the shape of the utility function (i.e., concavity over health) and preferences for less risky utility prospects (Segal and Spivak 1990; Wakker 1994). That is, individuals do not only care about their expected utility but also the utility distribution (Allais 1979; Hagen 1979; Quiggin 1982; Tversky and Kahneman 1991, 1992; Rabin 2000; Starmer 2000).

^4^
In line with Deaton (1997), we argue that I(V) should not be interpreted as an objective function policymakers should minimize. Instead, the expression should be seen as a statistical aggregator that provides information about the inequality in future health prospects that forces policy makers to think about how future health is distributed in relation to current income.

^5^
Equation (5) also links to discussions on ex‐post versus ex‐ante evaluation. Fleurbaey (2018) has argued that ex‐post evaluation in contexts with societal risk might be preferable over ex‐ante evaluation when the distribution of realized outcomes is known ex‐ante for each possible “state of the world.” In our setting, which focuses on individual health risk, the ex‐post versus ex‐ante distinction is less relevant as there are no “states of the world.” Every individual faces a distribution of future health prospects, but there is no common societal risk that affects each individual‐specific distribution. Nevertheless, a social planner who only cares about the covariance between current income rank and expected future health may neglect inequalities in dispersion but this is not the same as ex‐post evaluation under societal risk. Ex‐ante evaluation, in our setting, indicates that individual risk is taken into account by combining inequalities in expected future health with those in dispersion. This is a reasonable stance when the ex‐post distribution of realized future health outcomes is not known with certainty for each “state of the world” or when the social planner wants to account for individuals' current decision making being affected by facing dispersion about future health outcomes. For example, individual investment decisions might be distorted due to hedging against the full individual‐specific distribution of potential health outcomes (Picone et al. 1998; Chou et al. 2003).

^6^
Except for very specific cases, a given total amount of health cannot be concentrated in just the richest or poorest individual (or in the highest or lowest health quantile) when considering a bounded health variable (Erreygers 2009).

^7^
This property is known as the principle of income‐related health transfers. It was introduced by Bleichrodt and van Doorslaer (2006) and evaluates any rich‐to‐poor health transfer as desirable, including those transfers where the richer person has worse health. Experimental evidence on the principle's acceptability is mixed. In Bleichrodt et al. (2012), the majority of the respondents rejects the principle, while in Tarroux (2015) and K. I. Rohde et al. (2023) the majority instead accepts the principle, in particular when health is positively correlated with income.

^8^
We have chosen to not use the additional longitudinal data available to further model health dynamics over time. Our main purpose is to illustrate our new measurement apparatus. In addition, and as discussed in more detail in Section 5, using just two data points is also useful when less rich data is available.

^9^
This scale assigns a weight of 1 to the first adult and 0.5 to all other adults and 0.3 to all children (those aged under 15 years old).

^10^
The QALY weights are derived from the responses to the SF‐36 questionnaire using the SF‐6D preference‐based algorithm (Brazier et al. 2002). In order to combine morbidity and mortality into a single metric, health economists have elicited the value of particular health states by asking individuals to make decisions trading off quality versus length of life. QALY weights are derived for health states in comparison to full health which is given a QALY weight of one and death which is given a QALY weight of zero (see Drummond et al. 2015 for more details).

^11^
In practice one needs to make the bounds slightly larger than the true ones otherwise the transformed variable will be undefined as it will be equal to positive or negative infinity.

^12^
Asymptotically an infinite number of quantiles can be estimated but in a finite sample there will only be a finite possible number of quantiles with “different” estimated regressions (Portnoy 1991; Melly 2005). Also, when estimating the quantiles in the distribution's tails the uncertainty increases as the statistical power for these regressions are based on the support (size of the samples) on either side of the quantile.

^13^
The re‐transformed quantiles are always > 0.3 and < 1.001. One could choose values closer to the actual minimum and maximum (to further “stretch” the transformed continuous variables to negative and positive infinity) but this makes an extremely small difference to the final predicted quantiles.

^14^
Using 1000 draws yields expected conditional probabilities of being dead equal to the mortality rate at a one decimal precision (corresponding to 0.1% at most). Making more than 1000 draws (to further improve the precision) has a very minor impact on our results and increases the computational burden.

^15^
Age‐standardizing the index means that we are giving age special normative attention compared to the other predictors. This might be contestable in cases when age would be reflecting unfair variation in future health prospects (e.g., ‘unfair’ age discrimination), and not (only) the biological effect of age on survival. Since our empirical approach cannot distinguish between these channels, we assume that all variation in future health prospects due to age is fair, that is due to the biological effect of age on survival. In contrast to García‐Gómez et al. (2015) who use causal models to unravel fair and unfair drivers of health inequality, the predictive nature of our empirical approach impedes categorizing the other predictors into fair and unfair drivers of inequality (Fleurbaey and Schokkaert 2009).

^16^
When approximating Equation (10) by estimating a linear regression in each quantile, we introduce an error term as in the standard decomposition by Wagstaff et al. (2003). As the health quantiles are predicted using the same covariate set, these errors are close to negligible. For simplicity, we do not include the error term in Equation (10) or Equation (11).

^17^
This error will also capture the approximation error discussed in footnote 16.

^18^
This more generally holds for any bivariate inequality index. The magnitude and sign of the impact of non‐random measurement error in one dimension (say health) on any bivariate index cannot be known a priori, exactly because one also needs to know a priori how the measurement error correlates with the second dimension.

^19^
Figure A1 in appendix Appendix C illustrates that our mortality model accurately predicts survival by current income.

## APPENDIX A Inequality Impact of Mean Preserving Health Transfers

This appendix provides a generalized discussion of the inequality impact of health quantile changes. While the linear functional form is imposed on the income and quantile weights in the main text, here we only assume that the income weights sum to zero and are monotonically increasing with i, while the quantile weights are positive, sum to Q and are monotonically declining with q. We start the discussion with small health transfers such that the quantile weights of the unaffected quantiles do not change. Next, we generalize to larger health transfers.

### Appendix A.1. Small Transfers between Individuals

We start with the case where the transfers are sufficiently small such that they do not affect the associated weights of the other quantiles. Under this assumption, the individuals (d denoting donor and r denoting recipient) are confronted with $h_{r}^{1}\leq \text{…}\leq h_{r}^{q^{r}-1}\leq h_{r}^{q^{r}}+b\leq h_{r}^{q^{r}+1}\leq \text{…}\leq h_{r}^{Q}$ and $h_{d}^{1}\leq \text{…}\leq h_{d}^{q^{d}-1}\leq h_{d}^{q^{d}}-b\leq h_{d}^{q^{d}+1}\leq \text{…}\leq h_{d}^{Q}$ after transferring b units of health from quantile $q^{d}$ of individual d to quantile $q^{r}$ of individual r. This leads to the following inequality change.

(A1)
$$\Delta I=\frac{1}{nQ}\left\{\left[\sum_{i\neq r,d}\left(\sum_{q=1}^{Q}z_{i}w_{q}h_{i}^{q}\right)\right]+z_{r}\left[\sum_{q\neq q^{r}}w_{q}h_{r}^{q}+w_{q^{r}}\left(h_{r}^{q^{r}}+b\right)\right]+z_{d}\left[\sum_{q\neq q^{d}}w_{q}h_{d}^{q}+w_{q^{d}}\left(h_{d}^{q^{d}}-b\right)\right]\right\}$$

(A2)
$$\begin{array}{ll} & -\frac{1}{nQ}\left(\sum_{i=1}^{n}\sum_{q=1}^{Q}z_{i}w_{q}h_{i}^{q}\right)=\frac{b}{nQ}\left(z_{r}w_{q^{r}}-z_{d}w_{q^{d}}\right)\\ & =\frac{b}{nQ}\left\{\left[z_{r}-z_{d}\right]-\left[z_{r}\left(1-w_{q^{r}}\right)-z_{d}\left(1-w_{q^{d}}\right)\right]\right\} \end{array}$$

showing that the inequality change is partly driven by the impact via the expected health level of both individuals, that is $\left(b/nQ\right)\left(z_{r}-z_{d}\right)$, and partly by the impact that runs via the dispersion of the future health distribution, that is $\left(b/nQ\right)\left[z_{r}\left(1-w_{q^{r}}\right)-z_{d}\left(1-w_{q^{d}}\right)\right]$. The quantile weights do not matter for the first part, while the income weights matter for both.

For transfers between health quantiles of the same individual or between individuals with the same income weight, equation (A2) reduces to $z_{r=d}\left(b/nQ\right)\left(w_{q^{d}}-w_{q^{r}}\right)$. Thus, reducing (increasing) the dispersion of the future health distribution faced by a poor individual makes inequalities more pro‐poor (pro‐rich) and the opposite happens when reducing (increasing) the dispersion of the health distribution faced by a rich person (see columns 1 and 2 in Table A1). However, for transfers between individuals with different income income weights, the inequality change cannot always be unequivocally signed because of the interplay between the income and quantile weights in equation (A2). Table A1 presents an overview of all possible configurations of the income and quantile weights.

**TABLE A1:** Health quantile transfers and the direction of the inequality change ΔI.

	Same income		Richer donates			Poorer donates		
	$z_{r}=z_{d}$		$z_{r}<z_{d}$			$z_{r}>z_{d}$		
	Negative	Positive	Negative	Opposite	Positive	Negative	Opposite	Positive
	$\left(1\right)$	$\left(2\right)$	$\left(3\right)$	$\left(4\right)$	$\left(5\right)$	$\left(6\right)$	$\left(7\right)$	$\left(8\right)$
Same quantile $w_{q^{r}}=w_{q^{d}}$	=0	=0	<0	<0	<0	>0	>0	>0
Lower quantile donates $w_{q^{r}}<w_{q^{d}}$	>0	<0	<0 ^a^	<0	<0	>0	>0	>0 ^b^
Higher quantile donates $w_{q^{r}}>w_{q^{d}}$	<0	>0	<0	<0	<0 ^a^	>0 ^b^	>0	>0

^a^ ΔI>0 when the difference between the income weights is small and the difference between the quantile weights large.

^b^ ΔI<0 when the difference between the income weights is small and the difference between the quantile weights large.

When the donating individual is richer than the receiving individual (columns 3–5) ΔI will generally be pro‐poor. This is always the case when the two affected health quantiles are the same for both individuals $\left(w_{q^{r}}=w_{q^{d}}\right)$: as $V_{i}$ increases/decreases by the same absolute amount for the poorer/richer individual $\left(bw_{q^{r}}/Q=bw_{q^{d}}/Q\right)$, and since the poorest individual has a smaller income weight $\left(z_{r}<z_{d}\right)$, the inequality change must be pro‐poor.

When the quantile weights differ, health inequality becomes more pro‐poor in most cases, but with a few exceptions (see ‡ in columns 3 and 5 of Table A1). A pro‐rich change might occur when health is transferred from a rather bad health prospect of a poor person to an already good health prospect of a marginally poorer person ($z_{r}<z_{d}<0$ and $w_{q^{r}}<w_{q^{d}}$). Or equivalently when the health transfer involves two relatively rich individuals $\left(0<z_{r}<z_{d}\right)$ and the least rich receives health at a relatively low ranked quantile $\left(w_{q^{r}}>w_{q^{d}}\right)$. Thus, while a health transfer from rich to poor always induces a pro‐poor change in a setting without risk, this does not necessarily translate to a situation with risk.^20^ This is because transferring a lower (higher) valued unit of health from a rich (poor) donor to a rich (poor), but marginally poorer, receiver who puts higher (lower) value on this unit of health, will make the rich individuals $\left(z_{r}>0\right)$, as a group, better off than the poor $\left(z_{i}<0\right)$.

The scenario where the donating individual is poorer than the receiving individual in columns 6–8 is analogous, but reverse, to the second scenario in columns 3–5. In general, the sign reversals in both scenarios are more likely the larger the difference between the quantile weights and the smaller the difference between the income weights.

### Appendix A.2. Large Transfers between Different Health Quantiles of Different Individuals

The intuition underlying the discussion in section Appendix A1 also holds when the health transfer affects the quantile weights of the unaffected quantiles. Assume that the transfer moves the $q^{r}$th health quantile s quantiles up and that the $q^{d}$th health quantile moves u quantiles down. In other words, we are interested in the inequality‐impact of individual r facing $h_{r}^{1}\leq \text{…}\leq h_{r}^{q^{r}-1}\leq h_{r}^{q^{r}+1}\leq \text{…}\leq h_{r}^{q^{r}+s}\leq h_{r}^{q^{r}}+b\leq h_{r}^{q^{r}+s+1}\leq \text{…}\leq h_{r}^{Q}$ and individual d facing $h_{d}^{1}\leq \text{…}\leq h_{d}^{q^{d}-u-1}\leq h_{d}^{q^{d}}-b\leq h_{d}^{q^{d}-u}\leq \text{…}\leq h_{d}^{q^{d}-1}\leq h_{d}^{q^{d}+1}\leq \text{…}\leq h_{d}^{Q}$. The resulting change of the inequality index equals $\Delta I=\left(z_{r}/nQ\right)\left\{\left[\sum_{l=q^{r}}^{q^{r}+s-1}w_{l}\left(h_{r}^{l+1}-h_{r}^{l}\right)\right]+w_{q^{r}+s}\left(h_{r}^{q^{r}}+b-h_{r}^{q^{r}+s}\right)\right\}+\left(z_{d}/nQ\right)\left\{w_{q^{d}-u}\left(h_{d}^{q^{d}}-b-h_{d}^{q^{d}-u}\right)+\right.$ $\left.\left[\sum_{l=q^{d}-u+1}^{q^{d}}w_{l}\left(h_{d}^{l-1}-h_{d}^{l}\right)\right]\right\}$. This expression is more complex than Equation (A2), but the same intuition applies. The first term between the brackets is always non‐negative since $h_{r}^{l+1}\geq h_{r}^{l}$ and $h_{r}^{q^{r}}+b\geq h_{r}^{q^{r}+s}$; and the second term between brackets is always non‐positive as $h_{d}^{q^{d}}-b\leq h_{d}^{q^{d}-u}$ and $h_{d}^{l-1}\leq h_{d}^{l}$; just as $w_{q^{r}}$ is always positive and $-w_{q^{d}}$ always negative in Equation (A2).

## APPENDIX B Snapshot of the Underlying Regressions

Table A2 shows odds ratio estimates from the logit model in Equation (7) and from a subset of the logistic quantile regression in Equation (8). The predictions from these models are used to generate an approximation of the predicted future health distribution or individual health risk as described in Section 4.2.

**TABLE A2:** Regression results.

Explanatory variables (2011)	Mortality	Health conditional on survival				
	Logit odds ratios (std. errors)	Logistic quantile coefficients (std. errors)				
		5%	25%	50%	75%	95%
Health	0.083	2.459	3.353	3.691	2.840	2.643
	(0.030)	(0.196)	(0.109)	(0.098)	(0.107)	(0.197)
ln (equivalized household income)	0.878	0.099	0.158	0.122	0.108	0.045
	(0.074)	(0.039)	(0.025)	(0.024)	(0.022)	(0.034)
Age	1.009	−0.015	−0.007	−0.005	−0.006	0.006
	(0.022)	(0.007)	(0.005)	(0.004)	(0.004)	(0.009)
Age squared	1.00097	7.93E‐5	−4.31E‐5	−4.82E‐5	−3.19E‐5	−1.60E‐4
	(1.87E‐4)	(7.64E‐5)	(5.55E‐5)	(4.52E‐5)	(4.90E‐5)	(9.28E‐5)
Male	2.185	0.104	0.123	0.070	0.023	0.074
	(0.042)	(0.228)	(0.040)	(0.030)	(0.019)	(0.020)
Education (ref: Less secondary school)						
Secondary school completion	1.062	0.011	−0.028	−0.006	0.013	0.101
	(0.067)	(0.183)	(0.071)	(0.045)	(0.030)	(0.031)
Post school certificate or diploma	0.848	0.040	0.035	0.036	0.043	0.053
	(0.052)	(0.099)	(0.056)	(0.037)	(0.025)	(0.026)
University degree or higher	0.940	0.147	0.048	0.042	0.053	0.055
	(0.145)	(0.056)	(0.037)	(0.029)	(0.027)	(0.059)
Unemployed	0.913	−0.284	−0.180	−0.058	0.003	0.104
	(0.152)	(0.345)	(0.165)	(0.099)	(0.062)	(0.065)
Marital status (ref: Single)						
Married or living with partner	0.584	0.012	0.057	0.011	−0.021	−0.084
	(0.064)	(0.108)	(0.061)	(0.041)	(0.034)	(0.035)
Divorced/Separated/Widowed	0.771	−0.039	0.082	0.003	−0.073	0.011
	(0.093)	(0.153)	(0.089)	(0.059)	(0.046)	(0.044)
Smoking status (ref: Never)						
Smoker	2.515	−0.227	−0.100	−0.066	−0.036	−0.015
	(0.344)	(0.059)	(0.041)	(0.028)	(0.027)	(0.057)
Former	1.602	−0.059	−0.056	−0.036	−0.010	0.012
	(0.172)	(0.046)	(0.033)	(0.023)	(0.023)	(0.049)
Risky drinker	0.845	−0.005	−0.007	−0.015	0.006	0.043
	(0.198)	(0.103)	(0.058)	(0.038)	(0.048)	(0.111)
Country of birth (ref: Australia)						
Other English speaking country	0.761	−0.052	0.040	0.050	0.046	0.073
	(0.107)	(0.080)	(0.048)	(0.033)	(0.030)	(0.067)
Non‐English speaking country	0.687	−0.038	0.042	0.016	0.022	0.046
	(0.104)	(0.101)	(0.063)	(0.046)	(0.031)	(0.035)
Constant	0.014	−2.323	−2.652	−2.290	−1.173	−0.474
	(0.008)	(0.319)	(0.143)	(0.106)	(0.117)	(0.199)
Sample size (*N*)	12,833	9052	9052	9052	9052	9052

**TABLE A3:** Descriptives: Means by migrant group.

Variables	Aus born	Other OS Eng	OS non‐Eng
Health 2011	0.76	0.76	0.74
Health 2019	0.70	0.68	0.70
Equivalized household income ($‘000s per annum)	55.37	58.48	49.45
Age (years old)	46.35	52.95	48.23
Male	47%	51%	46%
Education			
Less than secondary school completion (reference)	30%	25%	19%
Secondary school completion	13%	13%	15%
Post school certificate or diploma	33%	33%	26%
University degree or higher	24%	28%	39%
Unemployed	3%	3%	3%
Marital status			
Single (reference)	18%	9%	12%
Married or living with partner	69%	75%	74%
Divorced/Separated/Widowed	14%	16%	14%
Smoking status			
Never (reference)	51%	43%	59%
Current	20%	18%	14%
Former	29%	39%	27%
Risky drinker	7%	5%	2%
Sample size	9783	1442	1608

*Note:* See notes of Table 1 for more details.

## APPENDIX C Model Prediction Performance

Figure A1 plots unadjusted sample and model predicted survival (with 95% confidence intervals) by current income quintiles. The results show that predicted survival by income quintile is very close to unadjusted sample survival, and that the confidence intervals around the model predicted mortality are very narrow. This confirms that mortality and its association with current income are accurately predicted by our mortality model.
